# Optimization of cultivation medium and cyclic fed-batch fermentation strategy for enhanced polyhydroxyalkanoate production by *Bacillus thuringiensis* using a glucose-rich hydrolyzate

**DOI:** 10.1186/s40643-021-00361-x

**Published:** 2021-01-30

**Authors:** Sarisha Singh, Bruce Sithole, Prabashni Lekha, Kugenthiren Permaul, Roshini Govinden

**Affiliations:** 1 Discipline of Microbiology, University of KwaZulu-Natal (Westville Campus), Durban, South Africa; 2 Biorefinery Industry Development Facility, Chemicals Cluster, Council for Scientific and Industrial Research, Durban, South Africa; 3 Discipline of Chemical Engineering, University of KwaZulu-Natal, Durban, South Africa; 4 Department of Biotechnology and Food Technology, Durban University of Technology, Durban, South Africa

**Keywords:** Polyhydroxyalkanoate, Glucose-rich hydrolyzate, Response surface methodology, Cyclic fed-batch fermentation, PHA productivity, Pulp and paper mill sludge

## Abstract

The accumulation of petrochemical plastic waste is detrimental to the environment. Polyhydroxyalkanoates (PHAs) are bacterial-derived polymers utilized for the production of bioplastics. PHA-plastics exhibit mechanical and thermal properties similar to conventional plastics. However, high production cost and obtaining high PHA yield and productivity impedes the widespread use of bioplastics. This study demonstrates the concept of cyclic fed-batch fermentation (CFBF) for enhanced PHA productivity by *Bacillus thuringiensis* using a glucose-rich hydrolyzate as the sole carbon source. The statistically optimized fermentation conditions used to obtain high cell density biomass (OD_600_ of 2.4175) were: 8.77 g L^−1^ yeast extract; 66.63% hydrolyzate (v/v); a fermentation pH of 7.18; and an incubation time of 27.22 h. The CFBF comprised three cycles of 29 h, 52 h, and 65 h, respectively. After the third cyclic event, cell biomass of 20.99 g L^−1^, PHA concentration of 14.28 g L^−1^, PHA yield of 68.03%, and PHA productivity of 0.219 g L^−1^ h^−1^ was achieved. This cyclic strategy yielded an almost threefold increase in biomass concentration and a fourfold increase in PHA concentration compared with batch fermentation. FTIR spectra of the extracted PHAs display prominent peaks at the wavelengths unique to PHAs. A copolymer was elucidated after the first cyclic event, whereas, after cycles CFBF 2–4, a terpolymer was noted. The PHAs obtained after CFBF cycle 3 have a slightly higher thermal stability compared with commercial PHB. The cyclic events decreased the melting temperature and degree of crystallinity of the PHAs. The approach used in this study demonstrates the possibility of coupling fermentation strategies with hydrolyzate derived from lignocellulosic waste as an alternative feedstock to obtain high cell density biomass and enhanced PHA productivity.
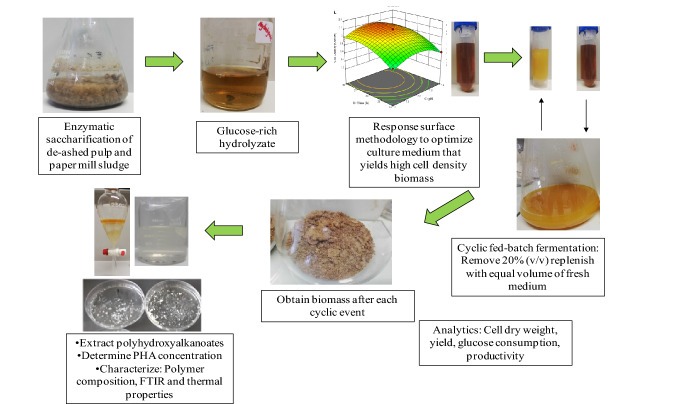

## Introduction

An estimated 240 million tons of plastics are produced globally every year. Most of these plastic materials are petroleum-based and are resistant to degradation resulting in accumulation and pollution of forestry and marine environments (Gholamveisi et al. 2018). With crude oil reserves dwindling, the dependence on exhaustible fossil fuel resources is unsustainable. This can be mitigated by the exploitation of biopolymers, i.e., natural polymers generated by living organisms, consisting of monomeric units that are covalently bonded to form larger molecules. Examples include polyhydroxyalkanoates (PHAs) such as polyhydroxybutyrate (PHB) and polyhydroxybutyrate–valerate copolymer (PHBV) that occur as intracellular granules produced by bacteria and are accumulated by the cell to act as carbon and energy storage reserves (Sheu et al. 2000; Sin et al. 2014). PHAs are considered to be carbon-neutral and environment-friendly. Research into PHAs has intensified since the discovery that PHA-bioplastics are non-toxic, biodegradable and that their mechanical, physical, and thermal characteristics are similar to fossil fuel-based plastics, such as polypropylene and polyethylene (Muhammadi et al. 2015). Thus, PHAs have the potential to replace fossil fuel-derived plastics. However, the PHA production cost is a significant problem as carbon source alone accounts for 45% of the total production cost, hindering the market growth of PHA-based plastics (Annamalai et al. 2017). For PHAs to be cost-competitive with petroleum-derived plastics, sustainable, renewable, and cheaper alternative carbon source substrates need to be explored. Furthermore, employing microbial strains capable of high cell density growth and PHA production in waste-derived cultivation media will also be beneficial.

Waste biomass is an attractive alternative source of fermentable sugars for application as inexpensive feedstock for the production of platform chemicals (Moritz and Duff 1996; Peters 2006; Prasetyo et al. 2010; Martin-Sampedro et al. 2011). Glucose-rich hydrolyzate obtained from corrugated cardboard was used as a nutrient source for yeast proliferation (García et al. 2015). Hydrolyzates obtained from enzymolysis of recycled paper sludge was used for the production of lactic acid by bacteria (Marques et al. 2008), primary clarifier sludge from kraft and low-yield sulfite pulping operations (Duff et al. 1994) and recycled paper sludge (Madrid and Díaz 2011; Schroeder et al. 2015) was used in bio-ethanol production.

Moreover, hydrolyzates from wheat bran (Annamalai and Sivakumar 2016), waste office paper (Annamalai et al. 2017), and wheat straw (Ferreira and Schlottbom 2016) were successful as alternative substrates for PHB production. *Bacillus* species have an innate ability to produce a variety of hydrolytic enzymes enabling them to metabolize complex residues and utilize a diverse array of carbon wastes (Rohini et al. 2006). Thus, there is a growing interest in exploring native *Bacillus* strains and their potential to use agro-wastes residues and hydrolyzates for PHA production. *Bacillus* strains such as *B. thuringiensis* B417-5 and *B. thuringiensis* strain IAM 12077 displayed PHB-producing capability when using hydrolyzates from agro-waste residues (Gowda and Shivakumar 2014; Thammasittirong et al. 2017).

To enhance product yields, high cell density cultivations and high productivity during fermentation should be achieved, particularly for intracellular compounds. The development of high cell density cultivation strategies fundamentally uses different types of bioreactors and cultivation strategies (Ibrahim and Steinbüchel 2010). One such strategy is cyclic fed-batch fermentation (CFBF) that was first described by Pirt (1974) for penicillin production and has since been applied for enhanced production of antibiotics (Lynch and Bushell 1995), recombinant proteins (Argyropoulos and Lynch 1997), and PHAs (Ibrahim and Steinbüchel 2010; Haas et al. 2017; Gahlawat and Srivastava 2018). The CFBF process entails the partial withdrawal of fermentation medium and subsequent re-filling with an equal volume of fresh fermentation medium. The chemical composition of the fermentation medium must be consistent throughout the "empty-and-fill" process. The CFBF strategy prevents increases in concentrations of toxic by-products and enhances cell growth to achieve high concentrations of final biomass and increased PHA yields (Ibrahim and Steinbüchel 2010; Gahlawat and Srivastava 2018).

This study reports the practicality of employing *Bacillus thuringiensis* in a CFBF cultivation using glucose-rich hydrolyzate derived from enzymatic saccharification of pulp and paper mill sludge as the sole carbon source. Firstly, a statistical optimization study was conducted to elucidate the optimal fermentation medium to produce high cell density biomass. The initial use of batch fermentation was to assist in determining kinetic parameters. Thereafter, the CFBF cultivation strategy was used to enhance cell density and PHA production by *B. thuringiensis*. A comparison between batch fermentation and CFBF was performed to evaluate PHA yield and productivity. Lastly, PHAs were extracted after each cycle and characterized to determine their composition and thermal stability and compared with commercial PHB and PHBV.

## Materials and methods

### Bacterial strain, growth, and storage conditions

*B. thuringiensis* DF7 (Accession no. KC020161), previously isolated from *Eucalyptus dunnii* wood chips obtained from a plantation in Durban, South Africa, was used. The bacterium was stored at 4 °C on nutrient agar for short-term maintenance and, 40% glycerol stock cultures were stored at − 80 °C for long-term storage.

### Hydrolyzate production

A glucose-rich hydrolyzate was extracted from dry de-ashed pulp and paper mill sludge (PPMS) as per the method outlined by Singh et al. (2021). Briefly, PPMS obtained from a prehydrolysis kraft and kraft (PHKK) pulping mill was de-ashed using acidic pretreatment.

A five-factor Box–Behnken design was used to optimize the conditions for the maximum recovery of glucose (g L^−1^) from dry de-ashed PPMS fiber. After model validation, the observed yield of glucose was 20.56 g L^−1^. The hydrolyzate contained trace amounts of xylose (2.62%) and mannose (0.89%) as well as low concentrations of toxins such as hemicellulose-derived acetic acid (0.25%), sugar-derived furans (1.06%), and lignin-derived phenols (0.58%).

### Cultivation medium for high cell density production

Biomass production was conducted using shake-flask cultivation in 25 mL stoppered conical flasks. The cultivation medium included: 1.5 g L^−1^ (NH_4_)_2_SO_4_, 0.2 g L^−1^ MgSO_4_·7H_2_O, 2.5 g L^−1^ NaCl, 1.5 g L^−1^ KH_2_PO_4_, and 1.5 mL L^−1^ trace element solution. The concentration of yeast extract and glucose-rich hydrolyzate and the medium pH were varied as per the model design (Table 2). All flasks were incubated at 37 °C in an orbital shaker (New Brunswick Innova 44, Eppendorf) at 200 rpm for the relevant times (Table 2). After incubation, a 1 mL aliquot was aspirated, and cell density was recorded as the optical density at 600 nm (OD_600_) using a spectrophotometer (Varian Cary 60 UV/Vis spectrophotometer; Agilent Technologies) against a blank of the 1 mL respective uninoculated cultivation medium.

### Statistical optimization of fermentation medium

For this study, a Box–Behnken design (BBD) was applied to elucidate the optimal cultivation medium to obtain high cell density biomass. The design determined the concentration of yeast extract, the concentration of glucose-rich hydrolyzate, incubation time, and pH (independent variables) to obtain maximum cell density and any interaction(s) between different combination(s) of variables. The four factors studied in the design were designated as X_1_, X_2_, X_3_, and X_4_, respectively, and prescribed at three different levels (Table 1). The design generated a total of 27 experimental runs.

**Table 1:** Independent variables with their respective coded values and levels used in the Box–Behnken design

Coded value	Independent variable	Level		
		Low	Middle	Upper
		− 1	0	+ 1
X_1_	Yeast extract (g L^−1^)	6	8	10
X_2_	Hydrolyzate (% v/v)	50	75	100
X_3_	Time (h)	18	24	30
X_4_	pH	7.0	7.2	7.4

The results from the experimental runs were analyzed and interpreted using Design-Expert software V 12 (StatEase). Based on the cell density response and interaction of the variables, a multiple regression analysis of independent variables was used, and a second-order polynomial was fitted to the response data obtained from the design.

The second-order polynomial equation generated was as follows:

$$Y= {\beta }_{0}+ {\beta }_{i}{X}_{i}+ {\beta }_{ij}{X}_{i}{X}_{j}+ {\beta }_{ii}{X}_{i}^{2},$$

where *Y* is the predicted response, *β*_0_ is the model constant, *β*_*i*_ is the linear coefficient, *β*_*ii*_ is the quadratic coefficient, *β*_*ij*_ is the interaction coefficient, and *X*_*i*_ is the independent variable. The statistically not significant parameters (*p* > 0.05) and their interactions were omitted from the equation.

#### Data analysis

The ANOVA test was applied to determine the statistical significance (*p* < 0.05) of the BBD. Adequacy of models was checked by analysis of *R*^2^ and the *R*^2^ adjusted. The capability and statistical significance of the model equation and the model terms were evaluated by the *F*-test (Cao et al. 2008). The *F*-value is also checked to find out the significance of all the fitted equations at a 5% level of significance (Hanrahan et al. 2007; Smitha and Pradeep 2017; Czyrski and Sznura 2019). To visualize the relationship between response and experimental levels of a statistically significant factor(s) and to determine the optimum conditions, the fitted equations were expressed as 3-dimensional (3D) surface plots. Surface plots were determined by holding the other independent parameters at a constant middle range value.

#### Model validation

To validate the predicted value, experimental trials were conducted to determine the observed value using the optimum values for variables given by the second-order polynomial equation, critical values, and response surface plots. After model validation, the statistically optimized medium was used for seed and inoculum development, as well as the cultivation medium for the batch and CFBF.

### Batch fermentation and CFBF

Based on the results obtained from statistical optimization of the cultivation medium, the medium was prepared and used in downstream processes. For the batch and CFBF, the pre-inoculum was developed in 10% (v/v) suspensions in stoppered conical flasks and incubated at 37 °C in an orbital shaker (New Brunswick Innova 44, Eppendorf) at 200 rpm; for the optimal incubation time to produce high cell density biomass as determined from the BBD. The batch and CFBF cultivations of *B. thuringiensis* were conducted in a 2 L stoppered conical flask containing 500 mL optimized cultivation medium. For the fermentation, flasks were incubated at 37 °C in an orbital shaker (New Brunswick Innova 44, Eppendorf) and maintained at 200 rpm. CFBF was initiated as described for batch fermentation; however, when glucose concentration had depleted to a limiting concentration (~ 8 g L^–1^), the batch cultivation was converted to nutrient feed cycle mode wherein 20% (v/v) of the fermentation medium was removed from the flask and replenished with an equal volume of fresh medium (having the same composition as the starting medium). Four cycles of repeated-batch cultivation were executed in this manner. At the end of the batch cultivation and after each cycle of the CFBF, the residual carbohydrate content (g L^−1^), cell biomass (cell dry weight) (CDW; g L^−1^), and PHA produced (g L^−1^) were determined. The PHAs extracted at the end of each cycle were confirmed by polymer characterization analyses. The batch and CFBF were conducted in duplicate on two separate occasions.

### PHA extraction

PHAs were recovered using solvent extraction followed by non-solvent precipitation, as per the methodology of Munir et al. (2015). The hypochlorite–chloroform dispersion method is a simple and rapid method routinely used to recover high yields of PHA from cell biomass with high purity (Kunasundari and Sudesh 2011; Aramvash et al. 2018). The bacterial biomass was harvested by centrifugation at 5752×*g* for 20 min to form a pellet that was washed twice with distilled water and lyophilized. The cell dry weight of the lyophilized cells was determined gravimetrically. Thereafter, the lyophilized cells were transferred into a conical flask to which 100 mL of 5% sodium hypochlorite and 100 mL of chloroform were added. The mixture was agitated in a rotary shaker (New Brunswick Innova 44, Eppendorf) at 300 rpm at 37 °C for 3 h. The suspension was transferred into a separating funnel and left to stand for 30 min to allow phases to separate viz., an upper phase containing hypochlorite solution, a middle phase containing non-PHA material (cell debris and undisrupted cells), and the bottom phase consisting of PHA solubilized in chloroform. The bottom phase was decanted into a beaker, and nine parts of methanol was added to precipitate the PHAs. Finally, the PHA-precipitate was dried by evaporation at 30 °C, resulting in white flakes or powder. The dry weight of the extracted PHAs was determined gravimetrically.

The percentage yield of PHAs was determined using the following equation:

$$\mathrm{Yield }\left(\mathrm{\%}\right)= \frac{\mathrm{ PHAs }(\mathrm{g})}{\mathrm{CDW }(\mathrm{g})} \times 100$$

The volumetric bacterial biomass productivity was determined using the following equation:

$$\mathrm{Productivity }\left(\mathrm{g }\,{\mathrm{L}}^{-1}{\mathrm{h}}^{-1}\right)= \frac{\mathrm{CDW }\left(\mathrm{g }\,{\mathrm{L}}^{-1}\right)}{\mathrm{Time }\left(\mathrm{h}\right)}$$

The volumetric PHA productivity was determined using the following equation:

$$\mathrm{Productivity }\left(\mathrm{g }\,{\mathrm{L}}^{-1} {\mathrm{h}}^{-1}\right)= \frac{\mathrm{PHAs }\left(\mathrm{g }\,{\mathrm{L}}^{-1}\right)}{\mathrm{Time }\left(\mathrm{h}\right)}$$

### High-performance liquid chromatography (HPLC)

In order to quantitatively determine glucose in the fermentation medium, samples were prepared using standard Technical Association of the Pulp and Paper Industry (TAPPI) method T 249 cm-09 and analyzed using HPLC.

### Fourier transform infrared spectroscopy (FTIR)

The chemical structure of the extracted PHAs and commercial PHB and PHBV (Sigma Aldrich) were analyzed by FTIR. The presence of functional groups representative of PHAs were observed in infrared spectra of the powdered samples recorded in the wavenumber range from 600 to 4000 cm^−1^ using a Perkin Elmer spectrophotometer (Jasco FTIR-6100).

### Polymer composition

Thermally assisted hydrolysis and methylation-gas chromatography (THM-GC) using pyrolysis/GC–MS (Py/GC–MS) in the presence of strong organic alkali, tetramethylammonium hydroxide (TMAH), was employed to qualitatively and quantitatively elucidate the polymer composition of the extracted PHAs and commercial PHB and PHBV (Sigma Aldrich) (Torri et al. 2014). The main peaks observed were identified by comparing the mass spectra with the NIST library database. The compounds were identified based on retention time and mass spectra, with only similarities ≥ 85% considered genuine fits. The polymer composition (mol%) of each PHA was calculated using the methodology of Martínez-Sanz et al. (2014):

$${H}_{\mathrm{a},\mathrm{ b, c}}\left(\mathrm{\%}\right)= \frac{\mathrm{area }{H}_{\mathrm{a},\mathrm{ b, c}}}{\mathrm{ area }{H}_{\mathrm{a}}+\mathrm{area }{H}_{\mathrm{b}}+\mathrm{area }{H}_{\mathrm{c}}} \times 100$$

where a, b, and c represent the monomers polyhydroxybutyrate (HB), polyhydroxyvalerate (HV), or polyhydroxyhexanoate (HHx), respectively.

### Thermogravimetric analysis (TGA) and differential scanning calorimetry (DSC)

Thermogravimetric analysis was used to analyze the thermal stability of the extracted PHAs and commercial PHB and PHBV (Sigma). TGA was conducted using a TGA Q5000 (TA Instruments) with a temperature range from 30 to 600 °C and a heating rate of 10 °C min^−1^ in a nitrogen atmosphere (N_2_ flow rate = 40 mL min^−1^). The initial degradation temperature (T_5%_) and maximum decomposition temperature (T_max_) are the temperatures at which 5% and 95% weight loss occurred and were determined by analyzing the TGA and derivative thermogravimetric (DTG) graphs, respectively (Pradhan et al. 2018).

A differential scanning calorimeter (DSC Q2000, TA Instruments) was used to perform DSC analysis, where 10 mg of sample was loaded in an aluminum pan and heated from − 10 to 200 °C at a heating rate of 10 °C min^−1^. A heating and cooling rate of 10 °C min^−1^ was used as well as a nitrogen environment with a gas flow of 20 mL min^−1^. The glass transition temperature (*T*_g_) was identified as the point of inflection in the thermogram between onset and offset temperatures. The melting temperature (*T*_m_) and melting enthalpy (∆*H*_m_) were determined from the peak temperature and area under the peak of an endothermic event in the second heating cycle, respectively. The crystallization temperature (*T*_c_) and crystallization enthalpy (∆*H*_c_) were determined from the peak temperature and area under the peak of an exothermic event in the cooling cycle, respectively. The degree of crystallinity (*X*_c_) was calculated by dividing ∆*H*_m_ by 146 J g^−1^ (melting enthalpy of 100% crystalline PHB) (Pradhan et al. 2018). TGA and DSC analysis were conducted in duplicate for each PHA.

### Statistical analysis

The effects of batch fermentation and each cycle of the CFBF on the yield of CDW and PHA as well as on polymer composition were determined using one-way ANOVA and SPSS (V 27.0). Where necessary, a Bonferroni post hoc analysis was conducted, and a *p* < 0.05 was considered as statistically significant.

## Results and discussion

### Statistical optimization of biomass fermentation medium

**Fig. 1:**
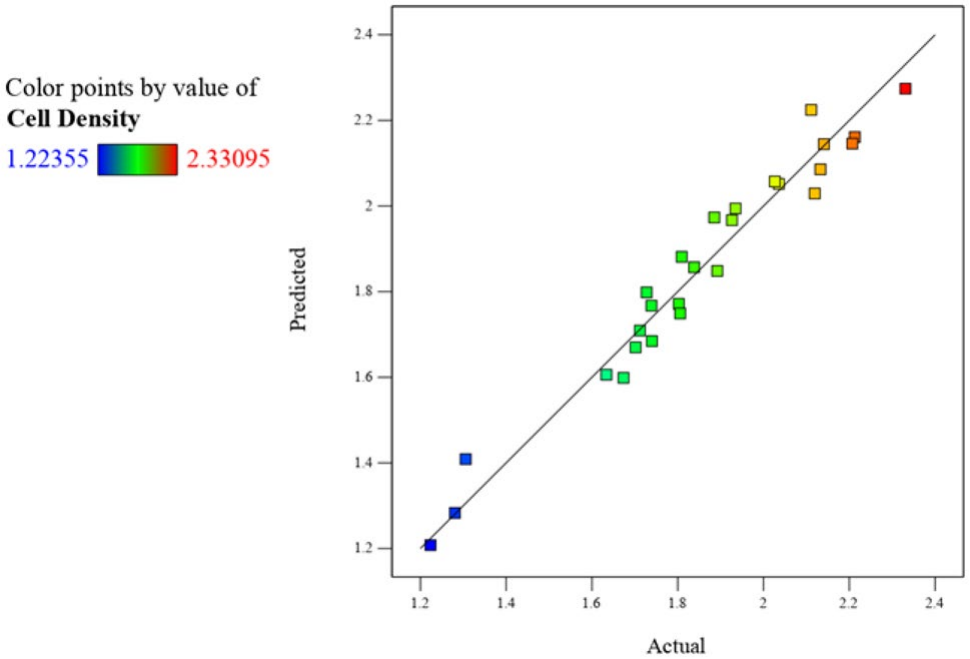
Graphical representation of the minimal difference between the actual (straight line) and predicted responses (squares) for the Box–Behnken design to obtain high cell density

A BBD with four factors, with replicated center points, was used for statistical optimization of the fermentation medium for the production of high cell density biomass. The parameters tested were; yeast extract (*A*), hydrolyzate concentration (*B*), incubation time (*C*), and pH (*D*) of the fermentation medium (Table 2). This model was used to determine the effects of individual or combined interactions of the four independent variables on the yield of biomass. The experimental conditions, batch runs, as well as the corresponding actual, predicted, and residual responses for biomass yield are represented in Table 2 and Fig. 1. An increase in cell density from OD_600_ of 1.8094 to 1.8923 was noted when the concentration of yeast extract decreased from 10 to 6 g L^−1^ (runs 10 and 11). An increase in the concentration of hydrolyzate from 50 to 100% negatively affected cell proliferation (runs 14 and 15), whereby cell density decreased from OD_600_ of 1.7275 to 1.2236. The effect of pH is observed in runs 23 and 25, where a pH of 7 (closer to neutral) favored high cell density production with an OD_600_ of 2.1408 noted compared with pH 7.4, where an OD_600_ of 1.8025 was observed. A longer fermentation time also favored high cell density production (runs 5 and 6), where an increase in cultivation time from 18 to 30 h increased cell density from OD_600_ 1.6736 to 1.9265 (Table 2). The highest OD_600_ of 2.3309 was noted for run 22 using the conditions: 10 g L^−1^ yeast extract; 75% hydrolyzate; incubation for 30 h; and a fermentation medium at pH 7.2. It was observed that the predicted and the actual experimental responses of biomass are comparable, with minimal difference noted between the data (Fig. 1). The minimal deviation from the straight line indicates less variation from the predicted value and is a satisfactory correlation between experimental data and predictive data, thus proving a high prognostic ability of the BBD (Tesfaye et al. 2018a).

**Table 2:** The actual, predicted, and residual responses for each experimental run using the Box–Behnken design for high cell density production

Experimental run	Variables				Cell density (OD_600_)		
	Yeast extract (g L^−1^)	Hydrolyzate (% v/v)	Time (h)	pH	Actual	Predicted	Residual
1	6	50	24	7.2	2.0359	2.0517	− 0.0158
2	10	50	24	7.2	2.1195	2.0293	0.0902
3	6	100	24	7.2	1.3056	1.4088	− 0.1032
4	10	100	24	7.2	1.7119	1.7091	0.0028
5	8	75	18	7.0	1.6736	1.5987	0.0749
6	8	75	30	7.0	1.9265	1.9670	− 0.0405
7	8	75	18	7.4	1.6336	1.6061	0.0275
8	8	75	30	7.4	1.8854	1.9733	− 0.0879
9	8	75	24	7.2	2.2129	2.1609	0.0519
10	6	75	24	7.0	1.8923	1.8377	0.0546
11	10	75	24	7.0	1.8094	1.8706	− 0.0613
12	6	75	24	7.4	1.8057	1.7384	0.0672
13	10	75	24	7.4	1.9349	1.9835	− 0.0486
14	8	50	18	7.2	1.7275	1.7876	− 0.0601
15	8	100	18	7.2	1.2236	1.1973	0.0262
16	8	50	30	7.2	2.0264	2.0466	− 0.0203
17	8	100	30	7.2	1.7399	1.6739	0.0660
18	8	75	24	7.2	2.1112	2.1349	− 0.0238
19	6	75	18	7.2	1.7387	1.7779	− 0.0392
20	10	75	18	7.2	1.8382	1.8676	− 0.0294
21	6	75	30	7.2	2.1328	2.09634	0.0364
22	10	75	30	7.2	2.3309	2.2847	0.0463
23	8	50	24	7.0	2.1408	2.1552	− 0.0144
24	8	100	24	7.0	1.2805	1.2938	− 0.0133
25	8	50	24	7.4	1.8025	1.7821	0.0203
26	8	100	24	7.4	1.7019	1.6804	0.0214
27	8	75	24	7.2	2.2071	2.2352	− 0.0281

The responses of predicted and experimental values were computed by means of ANOVA to determine the ability of the polynomial expression to predict the responses statistically. The ANOVA for the quadratic model of the response for cell density yield is presented in Table 3. The processed data from the experimental design enabled the calculation of the coefficients of the regression equation. The second-order polynomial equation is as follows:

$$ \begin{aligned} {\text{Response}}:{\text{Biomass}}\left( {{\text{OD}}_{600} } \right) & = + 2.18 + 0.0695A + 0.2408B + 0.0034C \\ & \quad + 0.1839D + 0.0807AB + 0.0530AC + 0.0247AD \\ & \quad + 0.1899BC + 0.0544BD - 0.0003CD - 0.0656A^{2} \\ & \quad - 0.2956B^{2} - 0.2117C^{2} - 0.1630D^{2} \\ \end{aligned}$$

**Table 3:** ANOVA for response surface quadratic model for biomass production by *B. thuringiensis*

Source	Sum of squares	Degrees of freedom	Mean square	*F*-value	*p*-value
Model	1.94	14	0.1384	16.13	< 0.0001**
*A*-yeast extract	0.0580	1	0.0580	6.75	0.0266*
*B*-hydrolyzate	0.6956	1	0.6956	81.04	< 0.0001**
*C*-pH	0.0001	1	0.0001	0.0162	0.9012
*D*-time	0.4058	1	0.4058	47.27	< 0.0001**
*AB*	0.0260	1	0.0260	3.03	0.1122
*AC*	0.0113	1	0.0113	1.31	0.2789
*AD*	0.0024	1	0.0024	0.2837	0.6059
*BC*	0.1443	1	0.1443	16.81	0.0021*
*BD*	0.0118	1	0.0118	1.38	0.2677
*CD*	3.306 × 10^–7^	1	3.306 × 10^–7^	0.0000	0.9952
*A*^2^	0.0230	1	0.0230	2.68	0.1329
*B*^2^	0.4661	1	0.4661	54.30	< 0.0001**
*C*^2^	0.2391	1	0.2391	27.85	0.0004**
*D*^2^	0.1417	1	0.1417	16.50	0.0023*
Residual	0.0858	10	0.0086		
*R*^2^	0.9576				
Adjusted *R*^2^	0.8982				

*Significant (*p* < 0.05) 5% level of significance

**Highly significant (*p* < 0.01) 1% level of significance

where *A*, *B*, *D*, *BC*, *B*^2^, *C*^2^, and *D*^2^ were the statistically significant model terms indicating confidence in the results for the parameters yeast extract concentration (*A*), hydrolyzate concentration (*B*), incubation time (*C*), and pH (*D*).

The mathematical model equation for the production of biomass is within the limits of the conditions tested. Analysis of the regression equation infers that maximum cell density production of 2.3309 at OD_600_ is within the selected factor ranges and is influenced by all of the independent variables either in linear or quadratic terms. When considered in linear terms, the concentration of hydrolyzate (*B*) and incubation time (*D*) showed the greatest influence on biomass production. This suggests that biomass production should be relatively insensitive to linear variation of the other parameters. When considered with respect to quadratic terms, the concentration of hydrolyzate, reaction time, and pH had positive significance on biomass production (Table 3). The only significant interactive effect of variables was between hydrolyzate concentration and time (BC) (*p* < 0.05), indicating that this strong interaction has a positive significance on yielding high cell density biomass (Table 3). The negative values of coefficients of the regression equation confirm that an increase in the value of any of the factors will decrease the biomass yield. The model *F*-value of 16.13 and *p* < 0.01 implies that the model is significant, and there is a 0.01% chance that an *F*-value this large can be due to noise. The ANOVA indicated that this regression model was highly significant (*p* < 0.01). The value of *p* > *F* < 0.001 implies that the model terms are significant and that there is less than a 1% chance that the observed values were due to chance. *R*^2^ or coefficient of determination is the proportion of variation in the response attributed to the model rather than to random error, and for a good fit of a model, *R*^2^ should be at least 80% (Cao et al. 2008). The suitability of the model was confirmed by a satisfactory *R*^2^ value of 0.9576, which means that 95.76% of the variability in the response could be explained by the model and that 5% of the variations occur while performing the experiments, thus indicating a realistic fit of the model to the experimental data (Table 3). The *R*^2^ coefficient measures the number of reductions in the variability of the response obtained using the independent factors within the model and confirms a satisfactory adjustment of the proposed model to the experimental data. The adjusted determination coefficient *R*^2^ value was 0.8982 indicating adequate signal, and this high value also supports that the model was highly significant. The coefficient of variation (CV) is the ratio of the standard error of the estimate to the mean value of the observed response, expressed as a percentage, and a model with a CV below 15% can be considered reasonably reproducible as it suggests higher reliability of the experiment and demonstrates greater reliability of the trials (Cao et al. 2008; Tesfaye et al. 2018b; Limkar et al. 2019). In the current study, the CV of 5.01% indicates a high degree of precision in the experiment. Adequate precision measures the signal-to-noise ratio, and a ratio greater than four is desirable. For the present BBD, a ratio of 14.501 indicates an adequate signal suggesting that this model can be used to navigate the design space.

**Fig. 2:**
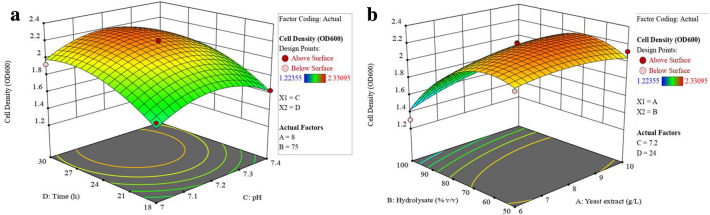
Three-dimensional (3D) response surface generated by the model for the independent variables that affect the yield of biomass; time and pH (**a**) and concentration of hydrolyzate and yeast extract (**b**)

The interactive effects between the response variable and the test variables are graphically illustrated as 3D-response surface plots generated by the model, as represented in Fig. 2. The elliptical or circular shape of contours in 3D plots helps to predict the major interaction between the variables (Tesfaye et al. 2018b). An elliptical contour plot is indicative of highly significant interactions between the variables. From these plots, it is evident that a pH of 7.1–7.2 and a cultivation time of ~ 27 h will result in high cell density (OD_600_ of 2.2). The cell density decreases at higher pH or decreased cultivation time (Fig. 2a). It is evident that yeast extract of ~ 8 g L^−1^ enhanced biomass production (Fig. 2b). Nitrogen is an absolute requirement for cell growth and is assimilated for the synthesis of amino acids glutamine and glutamate (Cheng et al. 2015). Yeast extract comprises the water-soluble components of the yeast cell and is the main nitrogen source for bacterial growth. It is rich in growth-stimulating compounds such as peptides, carbohydrates, salts, vitamins, and free amino acids. Therefore, it is a useful ingredient in media for the cultivation of microorganisms as it supports protein synthesis and cell growth, and reduces the consumption rate of the carbon source, and minimizes the accumulation of by-products (Cheng et al. 2015; Nguyen and Tran 2018). Ferreira and Schlottbom (2016) found that as little as 1 g L^−1^ of yeast extract was sufficient to obtain high yields of *B. sacchari*, whereas Andersen and Jayaraman (2003) obtained the maximum cell density of *B. thuringiensis* subsp. g*alleriae* when 19.7196 g L^−1^ was used in the cultivation medium.

A hydrolyzate concentration of ~ 65% allowed for a cell density of OD_600_ of 2.2, with concentrations above or below this range being less effective for yielding high cell density biomass (Fig. 2b). During the hydrolysis of cellulosic biomass, sugars are not the only by-product. Toxic cell inhibitors such as volatile organic acids, furfurals, and acid-soluble lignin are also released (Larsson et al. 1999). The inhibitory effect of phenolic and other aromatic compounds on microbial growth and product yield is variable. One possible mechanism is that phenolic compounds such as furfural and 5-hydroxymethylfurfural (5-HMF) interfere with the cell membrane by influencing its functions and changing its protein-to-lipid ratio (Peters 2006; Zhang et al. 2018). Two common detoxification procedures to eliminate microbial inhibitors include separation by adsorption of inhibitors using activated carbon and over-liming. Such detoxification methods significantly reduce the concentration of inhibitors, thereby having a positive influence on the growth of biomass. However, detoxification processes are time-consuming and expensive (Kucera et al. 2017). Yu and Stahl (2008) observed that diluting hydrolyzate to about 50% v/v was better for cell growth and that the dilution effect significantly reduced the toxicity of the hydrolyzate. In the present study, it is possible that when using 100% (v/v) hydrolyzate, the presence of toxic compounds negatively affected cell proliferation resulting in lower cell density. In order to achieve a concentration of 50 or 75% of hydrolyzate, the hydrolyzate was diluted with a basal salt medium. Therefore, it is plausible that the simple dilution of hydrolyzate reduced the concentration of cell inhibiting toxic compounds, thus making the medium more favorable for cell growth. Furthermore, previous enzyme screening assays revealed that *B. thuringiensis* used in the present study synthesizes ligninase (Govender 2013). Ligninases can degrade furfural, HMF, or change their composition or structure to less toxic forms, thereby detoxifying lignocellulosic hydrolyzates (Yang et al. 2018). Values outside this range showed low efficiency for biomass production, confirming that the range of the chosen variables in the experimental design was conducive to obtain high yields of cell biomass.

### Model validation

The model in the present study was validated by conducting experiments under the predicted conditions. Post-analysis of the design was used to determine the point prediction, and a mean cell density at OD_600_ of 2.28011 was predicted using the factors; 8.77 g L^−1^ yeast extract; 66.63% hydrolyzate (v/v); a fermentation pH of 7.18; and an incubation time of 27.22 h. After duplicate experimental trials on two separate occasions, a maximum OD_600_ of 2.4175 was attained. The difference between experimental and predicted values revealed a good correlation amongst the observed and predicted results, thus verifying the validity of the response model and the reality and accuracy of optimal conditions for the variables.

### Batch fermentation and CFBF

**Fig. 3:**
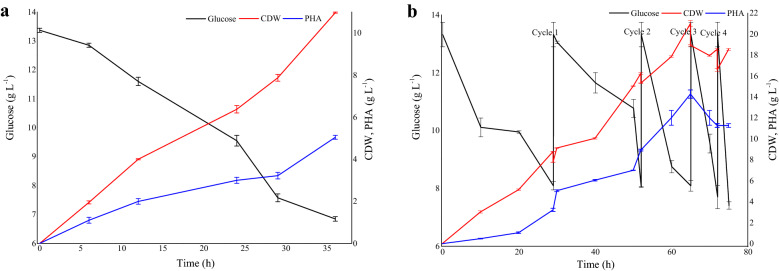
Kinetics of cell proliferation, carbon consumption and PHA production by *B. thuringiensis* in batch fermentation (**a**) and cyclic fed-batch fermentation (**b**)

Figure 3a depicts the time course of batch cultivation of *B. thuringiensis* for PHA production, and the kinetic parameters are presented in Table 4. CFBF cycles 2, 3, and 4 were observed to significantly affect the yield of biomass (CDW) and PHA yield (*p* < 0.05). During batch cultivation, the culture exhibited an initial lag phase of around 4 h, after which it grew exponentially, resulting in a biomass yield of 7.75 g L^–1^ (CDW) and 3.22 g L^–1^ of PHA in 30 h, resulting in a PHA productivity of 0.107 g L^–1^ h^–1^. The culture reached the stationary phase after 38 h. It was important to establish the batch growth and product kinetics since this established the basic kinetic parameters that were used for the CFBF for enhanced PHA formation. Wang and Liu (2014) found that wood extract hydrolyzate contributed to enhanced PHB production by *Burkholderia cepacia* in batch fermentation and obtained 16.8 g L^−1^ of PHB after 9 days. A 72 h batch fermentation of *Cupriavidus necator* using waste office paper hydrolyzate resulted in cell biomass, PHB production, and PHB content of 7.74 g L^−1^, 4.45 g L^−1^, and 57.52%, respectively (Annamalai et al. 2017). The CFBF cultivation of *B. thuringiensis* is depicted in Fig. 3b, and the kinetic parameters are presented in Table 4. The conversion of the batch fermentation to CFBF started at 29 h when the glucose concentration was 8.09 g L^−1^, resulting in the first cycle of removal and subsequent re-filling of an equal volume of fresh medium. The slight decrease in cell concentration observed during the cycles is due to the addition of fresh medium that diluted the remaining fermentation medium; however, it gradually increased with continuous cell proliferation. The second cycle occurred at 52 h, and the third cycle at 65 h. It was observed that the culture continuously maintained its metabolic activities and continued to produce PHA even after three cycles. Based on the observation of continuously high cell density and PHA yields and glucose consumption, a fourth cycle was initiated at 72 h. This however, was not successful, and the yields decreased in comparison with CFBF cycles 1–3. Therefore, three cycles were sufficient to successfully achieve high cell density and PHA yields using the CFBF strategy. At the end of the third CFBF cycle (after 65 h), cell biomass of 20.99 g L^−1^ was noted with a PHA concentration of 14.28 g L^−1^ resulting in a yield of 68.03%. This cyclic strategy yielded an overall PHA productivity of 0.219 g L^–1^ h^–1^. The CFBF resulted in an almost threefold increase in biomass concentration and fourfold increase in PHA concentration, respectively, as compared with batch cultivation (Table 4). During batch cultivations, nutrients such as carbon and nitrogen substrates become limited during the course of fermentation, particularly during the exponential growth phase, thus affecting the growth of the culture. Furthermore, initiating batch fermentation with very high concentrations of substrates in the cultivation medium stands a risk of substrate inhibition phenomenon. Therefore, CFBF could be a better option as it ensures a controlled feeding of the substrate and adequate nutrient availability during the entire cultivation, thereby curbing the potential problem of substrate inhibition. CFBF also eliminates time-consuming activities such as cleaning, filling, and sterilizing a bioreactor to initiate batch cultivation (Gahlawat and Srivastava 2018). To the best of our knowledge, to date, there are no reports on CFBF cultivation for biomass and PHA production by *B. thuringiensis.* Gahlawat and Srivastava (2018) used CFBF to increase PHB concentration and productivity by *Azohydromonas australica*. They observed that at the end of the 60 h fermentation, the biomass and PHB yield was 24.90 g L^−1^ and 18.79 g L^−1^, respectively, with a PHA productivity of 0.29 g L^−1^ h^−1^. Ibrahim and Steinbüchel (2010) also investigated the use of CFBF to achieve high biomass growth and PHB productivity by the thermophilic bacterium, *Chelatococcus* sp. MW10 in a 42 L bioreactor. After three cycles and 265 h fermentation, a cell density of 115 g L^–1^, PHB concentration of 13.6 g L^−1^ h^−1^, and PHB yield of 11.8% was noted. The CFBF strategy employed by Haas et al. (2017) found that *Cupriavidus necator* achieved a high PHB concentration of 3.1 g L^−1^ and reached a cell density of 148 g L^−1^, which yielded 76% PHB.

**Table 4:** Growth and product kinetic parameters of *B. thuringiensis* during the batch and cyclic fed-batch fermentation

Fermentation	Time (h)	Residual glucose (g L^−1^)	CDW (g L^−1^)	PHA (g L^−1^)	PHA yield (%)	Biomass productivity (g L^−1^ h^−1^)	PHA productivity (g L^−1^ h^−1^)
Batch	36	6.98	7.75	3.22	41.54	0.258	0.107
CFBF cycle 1	29	8.09	8.75	3.22	36.8	0.302	0.111
CFBF cycle 2	52	8.05	16.29	8.95	54.94	0.313	0.172
CFBF cycle 3	65	7.94	20.99	14.28	68.03	0.323	0.219
CFBF cycle 4	72	7.96	18.52	11.28	60.90	0.257	0.157

### FTIR characterization of PHAs

**Fig. 4:**
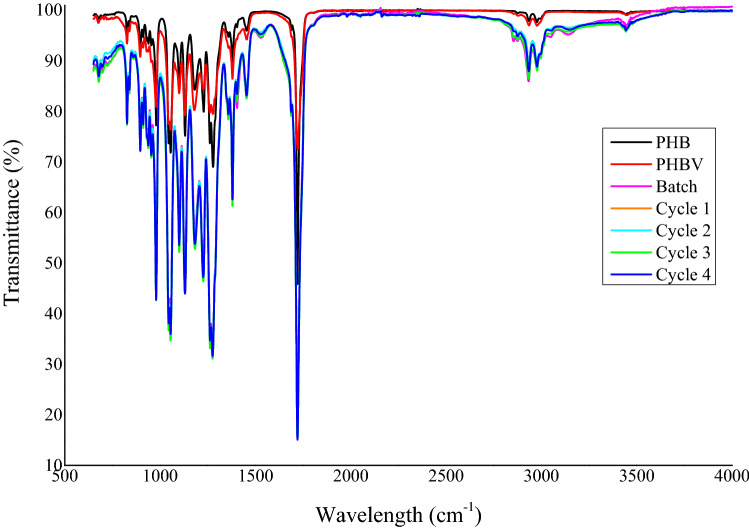
FTIR spectra of commercial PHB and PHBV compared with the PHAs extracted from *B. thuringiensis* after each cycle of the CFBF

FTIR was used to confirm PHA production by identifying the presence of functional groups present in PHA (Fig. 4). The peaks present at the ester, methylene, and terminal hydroxyl group are typically representative of the polymeric structure of PHAs (Apparao and Krishnaswamy 2015). The exact peak location and intensity are known to vary with the polymer chain length, concentration, and crystallinity of the PHA. The distinguishing peak of PHA is located around 1700–1738 cm^−1^ (C=O stretch) and a series of intense peaks located at 1000–1400 cm^−1^ (C–O stretch), which correspond to the ester group present in the molecular chain of highly ordered crystalline structures (Getachew and Woldesenbet 2016). The prominent pinnacle present at ~ 1708 cm^−1^ corresponds to ester carbonyl (C=O) extending the vibration of PHB (Fig. 4). Observation of adsorption bands around 1380, 1450, 2930, 1650, and 3400 cm^−1^ corresponds to –CH_3_, –CH_2_, CH, C–O, and O–H groups, respectively, which is typically present in pure PHB (Łabuzek and Radecka 2001). The strong peaks at 2923–2975 cm^−1^ are due to the C–H stretching methyl and methylene groups of alkanes, which are usually demonstrated by PHA polymers. The broad peak at 3396 cm^−1^ indicates the presence of O–H stretching of alcohol (terminal OH group) (Fig. 4) (Sindhu et al. 2016). The presence of copolymer like PHBV is denoted by a characteristic absorption peak in the region of 2933–2972 cm^−1^. Overall, the FTIR spectra of the extracted PHAs are comparable with commercial PHB and PHBV as they display prominent peaks at the wavelengths unique to PHA.

### Polymer composition

To identify the composition of the produced PHAs, Py/GC–MS and THM-GC were employed as these techniques permit accurate detection, characterization, and semi-quantification of PHA-monomeric moieties incorporated by the isolate (Torri et al. 2014; Baidurah et al. 2015). The variations in the polymer composition of PHAs extracted after batch and CFBF are highly significant (*p* < 0.01). Statistical analysis revealed that CFBF cycles 2, 3, and 4 significantly affected the mol% of HB and HV in the polymer (*p* < 0.05), whereas only CFBF cycles 3 and 4 significantly affected the mol% of HHx in the polymers (*p* < 0.05). It was surprising to observe that the commercial PHB used in the present study is not a PHB homopolymer as it contained 10.19% HV (Table 5). In the present study, it was observed that after the first cyclic event, a copolymer containing 62.61% HB and 37.39% HV was elucidated, whereas after CFBF cycles 2–4, the HB–HV–HHx terpolymer was noted (Table 5 and Additional file 1: Figures S1–S7). This differs from previous reports, which state that PHB was the predominant biopolymer synthesized when agro-waste-derived hydrolyzates from bagasse (Yu and Stahl 2008), wheat bran (Annamalai and Sivakumar 2016), rice straw (Sindhu et al. 2016), wood (Wang and Liu 2014), wheat straw (Ferreira and Schlottbom 2016), and waste office paper (Annamalai et al. 2017) were the sole carbon source in the fermentation.

**Table 5:** Polymer composition, thermal properties, and thermal degradation of PHA extracted from *B. thuringiensis* after each cycle of the cyclic fed-batch fermentation compared with commercial PHB, and PHBV

	Polymer composition (mol %)			Degradation temperature (°C)		Thermal properties						
	HB	HV	HHx	*T*_5%_	*T*_max_	*T*_g_ (°C)	*T*_m1_ (°C)	*T*_m2_ (°C)	∆*H*_m_ (J g^−1^)	*T*_c_ (°C)	∆*H*_c_ (J g^−1^)	*X*_c_ (%)
Batch fermentation	82.13	17.87	–	217.15	286.81	4.98	155.4	166.64	85.59	78.07	62.61	59
CFBF cycle 1	77.45	22.55	–	222.52	286.87	4.95	149.89	160.77	83.93	83.69	65.89	57
CFBF cycle 2	54.56	42.80	2.64	248.60	289.96	4.71	147.17	158.35	59.76	115.18	54.96	40
CFBF cycle 3	52.48	43.78	3.74	253	290.66	4.65	143.77	154.53	55.77	118.54	53.50	38
CFBF cycle 4	48.43	46.39	5.18	275.54	292.21	4.60	129.53	140.82	49.77	122.14	46.53	34
Commercial PHB	89.81	10.19	–	269.66	286.77	5.02	171.65	174.69	90.35	75.5	63.92	62
Commercial PHBV	43.35	56.65	–	264.04	288.32	4.85	138.22	146.94	60.55	113.17	58.06	41

### Thermal properties

#### TGA analysis

**Fig. 5:**
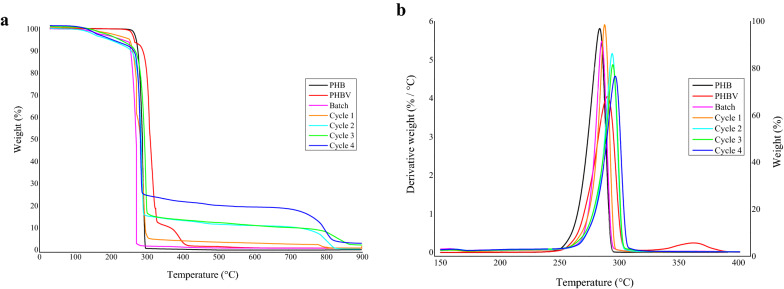
TGA (**a**) and DTG (**b**) thermograms of commercial PHB and PHBV, and the PHAs obtained after each cycle of the cyclic fed-batch fermentation

The thermal degradation profiles for commercial PHB and PHBV and the PHAs extracted from *B. thuringiensis* after each cyclic event are shown in Fig. 5. The initial (*T*_5%_) and maximum (*T*_max_) degradation temperatures for each sample are presented in Table 5 and Fig. 5. All of the samples displayed a similar thermal decomposition pattern, and the thermal degradation for all the samples occurs in a one-stage decomposition trend under a nitrogen atmosphere (Fig. 5a). The TGA curve shows that the weight loss (%) of all the samples occurs in two stages. In comparison with commercial PHB and PHBV, there is a noticeable difference in the initial and maximum thermal degradation. The initial degradation occurred in the range of 255.54–269.66 °C with a weight loss of ~ 1–8% of the total mass noted (Table 5; Fig. 5a). This weight loss results from the evaporation of physically adsorbed impurities or solvents like methanol, chloroform, etc., used during the extraction and separation processes (Pradhan et al. 2018). The maximum thermal degradation of the samples occurred around 286.77 °C to 292.21 °C, beyond the melting point of PHB (Table 5; Fig. 5b). The main mechanism of thermal decomposition of PHB corresponds to β-elimination of PHB chains that facilitate the formation of crotonic acid, dimeric, trimeric, and tetrameric volatiles (Vahabi et al. 2019). A shift in maximum degradation temperature of the PHAs is observed whereby *T*_max_ increased after each cyclic event compared with commercial PHB and PHBV (Table 5; Fig. 5b). Thus, it can be concluded that these PHAs have a slightly higher thermal stability compared with commercial PHB. The higher thermal stability of the extracted polymer could also be attributed to a more crystalline morphology (Pradhan et al. 2018). It is important to acknowledge high decomposition temperatures as it is a crucial factor for polymer processing in the industry since the material has to be tolerant and resist structural degradation, considering the high temperatures used for extrusion and injection molding to manufacture biodegradable films and molded pieces (Pradhan et al. 2018). Hassan et al. (2016) reported that the maximum thermal degradation for PHA obtained from *Bacillus* sp. 34 occurred between 237 and 320 °C, a range that was also observed in the present study. A similar observation was made for polymers obtained from *Alcaligenes* sp., where the maximum thermal degradation was between 220 and 315 °C (Melo et al. 2012). Pillai et al. (2017) found the thermal degradation of the PHB synthesized by *B. aryabhattai* to have an initial polymer degradation temperature at 247 °C and maximum degradation at 287 °C, which is similar to the degradation temperatures of the terpolymer PHA retrieved after the second cyclic event (Table 5). However, their standard PHB showed the initial and maximum degradations at 212 °C and 266 °C, respectively, which is substantially lower compared with the commercial PHB used in the present study (Table 5). The cyclic events resulted in PHAs having a noticeable shift in maximum thermal degradation temperature ranging from 250.77 to 262.56 °C, which is higher than the commercial PHB and PHBV, respectively. However, Sandhya et al. (2013) concluded that their sample contained many different hydroxyalkanoate monomers since they observed lower degradation temperatures compared to that of the standard PHB.

#### DSC analysis

**Fig. 6:**
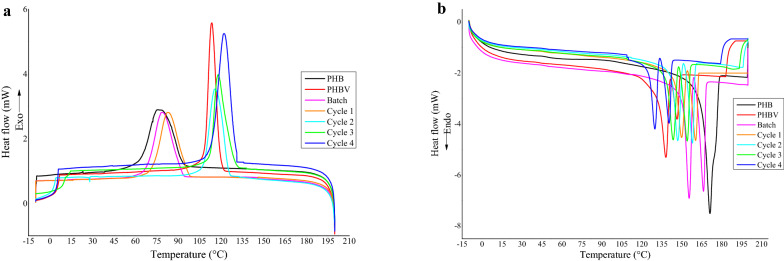
DSC thermogram displaying the cooling cycle (**a**) and the second heating cycle (**b**) used to characterize commercial PHB and PHBV, and the PHA extracted from *B. thuringiensis* after each cycle of the cyclic fed-batch fermentation

Differential scanning calorimetry analysis was conducted to elucidate the thermal transitions that a polymer undergoes as the sample is heated. The thermal transitions are manifested in terms of glass transition temperature (*T*_g_), melting point (*T*_m_), melting enthalpy (∆*H*_m_), crystallization temperature (*T*_c_), crystallization enthalpy (∆*H*_c_), and degree of crystallinity (*X*_c_) are summarized in Table 5. The DSC cooling and second heating cycle curves of commercial PHB and PHBV, as well as the PHAs, extracted from *B. thuringiensis* after each cyclic event, are shown in Fig. 6. The variations observed for *T*_g_, *T*_m_ *T*_c_, and *X*_c_ of PHAs extracted after batch fermentation and each cyclic event of CFBF are highly significant (*p* < 0.01). The *T*_g_ varied from 4.60 to 5.02 °C (Table 5). The structure of the polymer results in the initial movement of the polymer chain at a relatively lower temperature resulting in a decreased value of glass transition temperature (Pradhan et al. 2018). There was a wide variation in the *T*_c_ ranging from 75.5 to 118.54 °C (Table 5; Fig. 6a). Crystallization occurs due to the gain of mobilization of the polymer chains, which allows the polymer chains to organize and form crystals. The increasing *T*_c_ relates to the crystalline region in the polymer where the molecular chains are closely packed, and the secondary links are stronger in contrast to amorphous regions of a polymer. Therefore, high temperatures are required for the deformation of the polymer chain (Beber et al. 2018). PHAs with low *T*_c_ are problematic for melt-processing polymer procedures (Volova et al. 2013). The *T*_c_ for PHB synthesized from *B. megaterium* was ~ 113 °C, which is comparable to commercial PHBV analyzed in the present study (Chaijamrus and Udpuay 2008). The narrower crystallization peaks for PHBV and PHA from CFBF cycles 2, 3, and 4 observed in Fig. 6a are owed to the higher content of HV in the respective polymers and are also indicative of crystals with a more homogeneous size distribution. The larger crystallization peaks as observed for commercial PHB, PHA from batch fermentation and CFBF cycle 1 (Fig. 6a) are less desirable as they represent the formation of crystals with lower perfection and larger size distribution (Montanheiroa et al. 2016). In Fig. 6b, two distinct *T*_m_ endothermic peaks are observed for each sample, which is presented as a minor peak, followed by a dominant major peak at a higher temperature. For commercial PHB, the second peak appears as a shoulder peak of the main peak. The first *T*_m_ ranged from 129.53 to 171.65 °C, whereas the second *T*_m_ ranged from 140.82 to 174.69 °C (Table 5). The first melting peak originates from the melting of crystals formed during PHA sample preparation, and the second peak is due to the fusion of crystals formed during the heating phase of the DSC (Buzarovska and Grozdanov 2009). Other major contributors to the double melting behavior that results in multiple melting peaks include two different crystalline domains; the melting–re-crystallization–remelting mechanism; melting of different types of crystals with different sizes; different modifications; and thermal stabilities; or the melting of crystals with different lamellar thickness. Small and less perfect crystals melt at a lower temperature, and the larger and more perfect ones melt at a higher temperature (Wellen et al. 2015; Montanheiroa et al. 2016; Vahabi et al. 2019). Multiple melting peaks are a common feature of semi-crystalline polymers (Wellen et al. 2015). Multi-component PHAs, as observed in the present study, which have other alkanoate components or long side chains, tend to have a lower *T*_m_ (Saranya and Shenbagarathai 2011; Sandhya et al. 2013). Sharma et al. (2017) reported that the copolymer PHBV produced by *Pseudomonas putida* had a *T*_m_ of 137–170 °C, which is higher than the *T*_m_ of PHBV observed in the present study. The low *T*_m_ of the PHA characterized at CFBF cycle 4 is not unusual. Surendran et al. (2020) explain that incorporation of a minor quantity (5 mol%) of HHx can reduce the melting point to below 155 °C. The decreasing *T*_m_ in the present study is consistent with previous reports that had shown a decrease in melting temperature when the non-HB monomer fraction of the copolymer increased (Kehail et al. 2015). A low *T*_m_ is indicative of a polymer containing a high HV fraction (Surendran et al. 2020). A low *T*_m_ is also desirable as it implies that the polymer can be processed at a low temperature, making the polymer suitable for soft products such as films with improved ductility and flexibility (Liu et al. 2019). The presence of a pronounced gap between *T*_m_ and *T*_max_ observed in the present study (Table 5) is an important favorable characteristic of a polymer. It is indicative of polymers with a wide melt-processing window, thereby increasing the range of products (films, fibers, hollow forms, etc.,) that can potentially be produced from the polymer, as well as increasing the range of processing methods the polymer can withstand (solution spinning, extrusion, injection molding, etc.,) (Volova et al. 2013). The degree of crystallinity (*X*_c_) is one of the most important characteristics among the different mechanical performance and processability properties of the polymer and is summarized in Table 5. To minimize polymer processing challenges, *X*_c_ should ideally be ≤ 50%; otherwise, corrective actions are required resulting in challenges during polymer processing that can increase the operation price (Rodrigues et al. 2019). It was observed that the *X*_c_ for the cyclic events decreased from 52% at CFBF 1 to 34% after CFBF 4 (Table 5). Thus, the synthesized PHAs are less crystalline compared with commercial PHB and PHBV. Chaijamrus and Udpuay (2008) reported *X*_c_ = 60% for PHA extracted from *B. megaterium*, resulting in a highly crystalline polymer. That *X*_c_ is substantially higher compared with the *X*_c_ observed for PHAs in the present study. The *X*_c_ of the PHAs extracted from *B. thuringiensis* are lower than that of the commercial PHB and PHBV (Table 5). The decreasing *X*_c_ observed for PHAs from CFBF cycles 2, 3, and 4 is attributed to the presence of HHx specifically, as the incorporation of HHx in the polymer chain reduces the crystallinity of the PHA (Surendran et al. 2020). The *X*_c_ for PHBV obtained after batch fermentation, and CFBF cycle 1 is 59% and 57%, respectively, which is a relatively high crystalline PHBV copolymer. In addition, the *X*_c_ for the PHBV copolymers observed in the present study is similar to the *X*_c_ of commercial PHB (62%) than the *X*_c_ of commercial PHBV (41%) (Table 5). This observation is explained by Shang et al. (2012), whereby a high HV content in the polymer, as observed in commercial PHBV, decreases the crystallinity of a polymer. Surendran et al. (2020) further explain the isodimorphism behavior phenomenon that occurs exclusively in PHBV copolymers. When the HV fraction of the copolymer is < 37%, the HV lattice can co-crystallize into the same lattice as HB. The properties of the resultant polymer are similar to PHB, such as high crystallinity. A polymer with high crystallinity is stiff and brittle in nature (Singh et al. 2015; Możejko-Ciesielska and Kiewisz 2016). Therefore, a PHA with low crystallinity, such as the terpolymer obtained after CFBF cycles 2, 3, and 4, is advantageous as it is less brittle, thus increasing the range of applications of the polymer (Chaijamrus and Udpuay 2008). Furthermore, incorporating HV and HHx monomers into PHAs as well as having a combination of a low *T*_g_ and a low *X*_c_, as observed for PHAs in the present study, are favorable (Table 5). These characteristics change the mechanical performance of the polymer as it imparts elastomeric behavior rendering flexible polymers suitable for medical applications (Rai et al. 2011). In the present study, TGA and DSC analysis aided in highlighting the critical role that incorporation of non-HB units into the polymer chain plays for the enhancement of physical properties of PHA polymers.

## Conclusions

This study assessed the ability and efficiency of *B. thuringiensis* to assimilate glucose-rich hydrolyzate produced by enzymatic saccharification of PPMS. The isolate was able to use this hydrolyzate as sole carbon source for cell proliferation and PHA production. The statistical optimization using BBD aided in determining an optimal cultivation medium to produce high cell density biomass and also determined the success of the CFBF strategy. The CFBF strategy proved successful for simultaneously achieving high cell density and enhancing PHA production by *B. thuringiensis*. Three cycles of culture broth removal and subsequent re-filling with fresh medium at 29 h, 52 h and 65 h resulted in a maximum PHA yield of 14.28 g L^–1^ with the overall productivity of 0.219 g L^–1^ h^–1^. The present work demonstrated a threefold increase in PHA yield and a fourfold increase in productivity compared with batch cultivation. The isolate was a good producer of co- and ter-polymers. However, the polymer composition, and thermal properties varied after each cyclic event. On the basis of proof of concept, this study exhibits encouraging results. A range of very cheap raw materials are available for industrial PHA production, and coupled with the practicality of this strategy, it has potential to be scaled-up to pilot level for techno-economic feasibility analysis.

### Supplementary Information

**Additional file 1: Figure S1.** Pyrogram of commercial PHB. **Figure S2.** Pyrogram of commercial PHBV. **Figure S3. **Pyrogram of PHA extracted after cycle 1 of cyclic fed-batch fermentation. **Figure S4. **Pyrogram of PHA extracted after cycle 2 of cyclic fed-batch fermentation. **Figure S5.** Pyrogram of PHA extracted after cycle 3 of cyclic fed-batch fermentation. **Figure S6.** Pyrogram of PHA extracted after cycle 4 of cyclic fed-batch fermentation.

## Data Availability

All data generated or analyzed during this study are included in the manuscript.

## Abbreviations

PHA: Polyhydroxyalkanoate
PHB: Polyhydroxybutyrate
PHBV: Polyhydroxybutyrate–valerate copolymer
CFBF: Cyclic fed-batch fermentation
HHx: Polyhydroxyhexanoate
rpm: Revolutions per minute
BBD: Box–Behnken design
3D: 3-Dimensional
CDW: Cell dry weight
HPLC: High-performance liquid chromatography
TAPPI: Technical Association of the Pulp and Paper Industry
FTIR: Fourier transform infrared
THM-GC: Thermally assisted hydrolysis and methylation-gas chromatography
Py/GC–MS: Pyrolysis/gas chromatography–mass spectroscopy
TMAH: Tetramethylammonium hydroxide
TGA: Thermogravimetric analysis
DSC: Differential scanning calorimetry
*T*_g_: Glass transition temperature
*T*_m_: Melting temperature
*∆H*_m_: Melting enthalpy
*T*_c_: Crystallization temperature
∆*H*_c_: Crystallization enthalpy
*X*_c_: Degree of crystallinity
OD_600_: Optical density at 600 nm
