# Supplementary material for: Optimization of cultivation medium and cyclic fed-batch fermentation strategy for enhanced polyhydroxyalkanoate production by Bacillus thuringiensis using a glucose-rich hydrolyzate
Source: Bioresour Bioprocess. 2021 Jan 30;8(1):11. doi: 10.1186/s40643-021-00361-x (PMC10992944; doi:10.1186/s40643-021-00361-x)
Supplement: Supplementary file 1 — Additional file 1: Figure S1. Pyrogram of commercial PHB. Figure S2. Pyrogram of commercial PHBV. Figure S3. Pyrogram of PHA extracted after cycle 1 of cyclic fed-batch fermentation. Figure S4. Pyrogram of PHA extracted after cycle 2 of cyclic fed-batch fermentation. Figure S5. Pyrogram of PHA extracted after cycle 3 of cyclic fed-batch fermentation. Figure S6. Pyrogram of PHA extracted after cycle 4 of cyclic fed-batch fermentation. [file 40643_2021_361_MOESM1_ESM.docx]

Figure S1: Pyrogram of commercial PHB

Figure S2: Pyrogram of commercial PHBV

Figure S3: Pyrogram of PHA extracted after batch fermentation

Figure S4: Pyrogram of PHA extracted after cycle 1 of cyclic fed-batch fermentation

Figure S5: Pyrogram of PHA extracted after cycle 2 of cyclic fed-batch fermentation

Figure S6: Pyrogram of PHA extracted after cycle 3 of cyclic fed-batch fermentation

Figure S7: Pyrogram of PHA extracted after cycle 4 of cyclic fed-batch fermentation
